# Efficacy of Early Exposure Medical Programs for Underrepresented Students: A Systematic Review

**DOI:** 10.7759/cureus.109396

**Published:** 2026-05-21

**Authors:** Nikhil Jaganathan, Rithwik Guntaka, Varun Goel, Nadine Odo, Zhuo Sun, Mary E Arthur

**Affiliations:** 1 Anesthesiology and Perioperative Medicine, Medical College of Georgia, Augusta University, Augusta, USA

**Keywords:** early exposure, medical education, mentorship, premedical, underrepresented in medicine (urim)

## Abstract

Background: Despite decades of initiatives, the physician workforce does not reflect the United States demographic diversity. Underrepresented students are lost at multiple stages of the path to medicine due to academic, financial, and psychosocial barriers, including limited access to ethnoracially concordant role models. Although “pipeline” and “outreach” programs have been implemented, evidence-based evaluation of their effectiveness remains limited.

Objective: To identify components of early medical exposure programs associated with increased interest in medicine, self-efficacy, and diversity in the medical field.

Methods: We conducted a systematic review of published studies evaluating early medical exposure programs measuring quantitative outcomes. Searches of PubMed, Cochrane, and ScienceDirect were performed for publications from April 1999 to October 2025. Two reviewers independently screened studies, with discrepancies resolved by consensus.

Results: Of 464 studies identified, 24 met inclusion criteria. Mentorship (n=18) and research (n=13) were the common program components, followed by application preparation, observership experience, and subspecialty exposure. High school students were the primary participants (n=14), followed by undergraduates (n=8). Program duration varied from one day to three years, with longer programs associated with improved academic performance and increased higher education. Frequently reported outcomes included further education (n=10), increased biomedical and STEM degrees (n=8), increased interest in medicine (n=7), improved academic performance (n=4), and greater self-efficacy (n=4). Risk of bias was rated as “Low” or “Moderate.”

Conclusion: Early exposure programs for underrepresented students are associated with increased interest in medicine, improved self-efficacy, reduced perceived barriers, and academic benefits. Mentorship and research emerged as the most prominent components, associated with increased likelihood of further education and the pursuit of biomedical/STEM degrees.

## Introduction and background

Despite decades of initiatives, the physician workforce in the United States does not adequately reflect the nation’s demographics. This disconnect is a critical issue because a healthcare workforce that mirrors its community can more effectively serve patients and reduce health disparities. Studies have consistently shown that physicians from underrepresented in medicine (URiM) backgrounds are more likely to serve in medically underserved communities. Furthermore, ethnoracial concordance between physicians and their patients has been shown to improve patient compliance, trust, communication, and health outcomes [1,2]. Moreover, enhanced physician cultural competence may improve patient care for minority groups, thereby mitigating health disparities [2].

Although there is strong evidence of minority representation in medicine, medical school applicants from URiM groups do not fully reflect population diversity [1]. This phenomenon has been described as a “leaky pipeline,” in which underrepresented students are lost at various stages on the path to a medical career [1]. These leaks have been linked to intersectional barriers, including persistent academic gaps (often stemming from under-resourced K-12 education) and reliance on standardized metrics such as the Medical College Admissions Test (MCAT) and grade point average (GPA), where score gaps persist due to a lack of academic resources. Financial barriers are also a factor; the high cost of medical school, undergraduate debt for first-generation college students, and the inability to pay for test-preparation materials and application fees disproportionately affect URiM students [1]. Psychosocial factors, such as a lack of faculty diversity and ethnoracially concordant role models, can contribute to feelings of imposter syndrome and a perceived lack of belonging, which can deter students from persisting and hinder their self-appraised efficacy [1].

To address these challenges, institutions have begun implementing targeted “pipeline” or “outreach” programs designed to reach URiM students and provide support and career development in healthcare. Common interventions include hospital-based initiatives and university-led exposure programs [3]. Despite these efforts, a significant gap remains in the literature on a comprehensive analysis of structure, core components, and overall efficacy. This systematic review analyzes published literature on early medical exposure programs targeting students from middle school through high school, undergraduate and post-baccalaureate levels. By synthesizing data on program structures, curricula, target participants, and efficacy metrics, we aim to identify key takeaways regarding the components of such programs that best increase interest in medicine, build self-efficacy, and improve diversity in the medical field.

## Review

Methods

Data Sources and Search Strategy

We conducted a systematic review of published articles and abstracts on programs that provide early medical exposure to middle school, high school, undergraduate, and post-baccalaureate students. Two reviewers independently screened the literature for accuracy, with discrepancies resolved by consensus. Our review was derived from PubMed, Cochrane, and ScienceDirect databases from April 1999 to October 2025, including retrospective analyses, cohort studies, prospective surveys, and thematic analyses with quantitative data.

Inclusion and Exclusion Criteria

Our inclusion criteria involved specific search terms and Medical Subject Headings (MeSH) - “underrepresented,” “minority,” “mentorship,” “undergraduate,” “high school,” “URM,” “UriM,” “early exposure,” “program,” “medicine,” and “premedical” - to capture literature grounded in well-established frameworks addressing pathway development and workforce diversity initiatives in medicine [4-28]. After careful review, we eliminated duplicate and similar studies. Our exclusion criteria included secondary literature (e.g., systematic reviews, commentaries), studies lacking quantitative outcome measures (e.g., interest in medicine, academic performance, perceived barriers), and studies exclusively evaluating graduate or residency medical education initiatives.

Literature Screening and Data Extraction

Full-text English publications were analyzed by program type, participants, methodology, notable results, and conclusions. Our review included primary studies of programs that provide early medical exposure to middle school, high school, undergraduate, or post-baccalaureate students from at least one underrepresented racial, ethnic, gender, or socioeconomic group. We analyzed the structure, curriculum, target participants, and program efficacy metrics of the early exposure programs. Common qualitative endpoints from the included literature were extracted and categorized to assess outcomes and efficacy.

Risk of Bias

The Risk of Bias in Non-randomized Studies of Interventions, (ROBINS-I) V2 scale was used to assess risk of bias. Each of the 24 studies was evaluated across seven bias domains: confounding, participant selection, classification of interventions, deviations from intended interventions, missing data, measurement of outcomes, and selection of the reported results. Risk of bias, categorized as “Low,” “Moderate,” “Serious,” or “Critical” for each domain [29], was found to be either “Low” or “Moderate” in this analysis. Bias was “Moderate” almost universally for domains 1 and 2, likely due to the difficulty controlling for confounding variables in the qualitative endpoint analyses, intersectional barriers, and the nature of programs targeting a non-random URiM-focused student population. This assessment was first made independently by two authors and discussed with another author to resolve discrepancies by consensus.

Quality Assessment

The studies that met the inclusion criteria were also screened by the Oxford Levels of Evidence criteria [30]. Studies below Level of Evidence of 2C were excluded. This meant that we analyzed only outcome studies or cohort studies, without including case-control, case-series, case reports, or expert opinions, in order to ensure a high quality of data. The assessments were made by two reviewers with high inter-rater reliability.

Results

**Figure 1:**
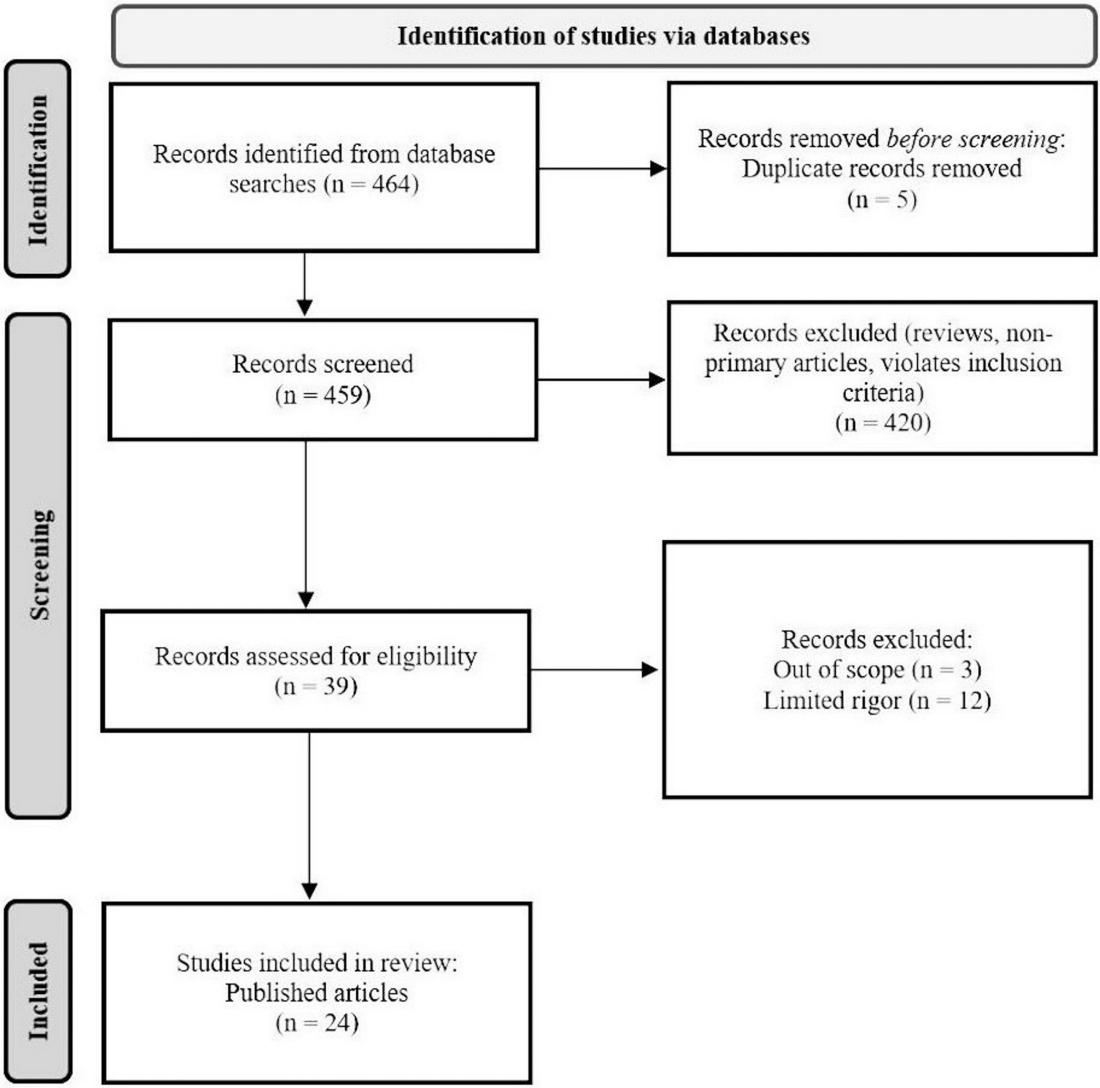
Flow diagram of the selection process

After careful review, 24 English-language full-text publications were included in the final analysis (Figure 1).

Literature Search

A total of 464 studies were identified from the databases searched, and 459 were selected for screening after duplicates were removed. After reviewing the abstracts, 420 manuscripts were excluded because they were reviews, non-primary articles, or did not meet the inclusion criteria. The remaining 39 manuscripts underwent full-text review and were evaluated for scope and methodological rigor, resulting in the exclusion of 15 articles. A total of 24 studies met the inclusion criteria and were retained for synthesis in accordance with the Preferred Reporting Items for Systematic Reviews and Meta-Analyses (PRISMA) guidelines (Figure 1) [4-28]. We verified that these studies were not redundantly represented in prior systematic reviews.

Characteristics and Methodological Quality of Included Studies

We included primary studies of programs that offer early medical exposure to middle school, high school, undergraduate, or post-baccalaureate students from underrepresented groups in medicine. We sought studies that reported quantitative and comparable outcome measures, such as interest in medicine following program intervention. Table 1 summarizes the study methodology, participants, notable results, and conclusions [4-27]. Table 2 provides the risk-of-bias designations for the included studies.

**Table 1:** Summary of pipeline and early exposure medical programs CSM: Careers in Science in Medicine; HPPI: Health Professions Partnership Initiative; JHU: Johns Hopkins University; HS: High school; LLU: Loma Linda University; MCPHS: Massachusetts College of Pharmacy and Health Sciences; MEDP: Medical Education and Development Program; MMP: Medical Mentorship Program; MS: Middle school; NC-DIPP: North Carolina Diversity and Inclusion Pathway Program; NIH: National Institutes of Health; PB: Post-baccalaureate; RISE: Research Internship and Science Education; ROEO: Reach One Each One Program; SHPEP: Summer Health Professions Education Program; MYSP: Stanford Medical Youth Science Program; UC: University of California; SREP: Summer Undergraduate Research Education Program; SUConn: University of Connecticut; UCSD: University of California San Diego; UG: Undergraduate; UMich: University of Michigan; UNC: University of North Carolina

Study	Program & Duration	Study Design	Population	Key Outcomes
Afghani et al. [4]	UC Irvine Summer Premed Program (2 weeks)	Cross-sectional survey	HS	High satisfaction; improved leadership skills for mentors
Bergstein et al. [5]	JHU Zoom panel	Cross-sectional survey	UG	↑ interest in orthopedics; ↓ perceived gender barriers
Crews et al. [6]	JHU CSM Initiative (1 week–2 years)	Retrospective cohort	HS/UG/PB	High college/STEM entry; strong graduate school progression
Danner et al. [7]	ROEO (11 weeks)	Cross-sectional survey	HS	High college enrollment; strong pre-med participation
Douse et al. [8]	Mayo SREP (10 weeks)	Cross-sectional survey	UG	↑ satisfaction; research output
Giordani et al. [9]	UMich PB (1 year)	Comparative cohort	PB	Medical school competence achieved
Goldsmith, Tran, and Tran [10]	MCPHS exposure (1 day)	Cross-sectional survey	MS	↑ health professions knowledge and interest
Hutchings et al. [11]	Henry Ford mentorship (up to 7 weeks)	Cross-sectional survey	HS	↑ confidence and surgery interest
Krecko and Jung [12]	Doris Duke programs	Cross-sectional survey	HS	High STEM participation
Papp et al. [13]	Alberta camp (1 week)	Cross-sectional survey	HS	Improved knowledge
Rivers et al. [14]	NIH STEP-UP (8-10 weeks)	Cross-sectional survey	HS/UG	High satisfaction; universal project completion
Robinson et al. [15]	NC-DIPP (1 year)	Cross-sectional survey	UG	Strong mentorship outcomes
Rohrbaugh and Corces [16]	Emory RISE (3 years)	Retrospective cohort	HS	High college/STEM progression
Salto et al. [17]	LLU program (8 weeks)	Cross-sectional survey	HS/UG	↑ research self-efficacy
Satpathy et al. [18]	UCSD program (8 weeks)	Cross-sectional survey	HS	High STEM interest, graduate school application rates
Schneid et al. [19]	UCSD prematriculation	Retrospective cohort	PB	Improved academic outcomes
Stephenson-Hunter et al. [20]	Einstein program (6 weeks)	Mixed-methods	UG	↑ self-efficacy
Stewart et al. [21]	UConn fellowship (7 weeks)	Retrospective cohort	UG	High undergraduate graduation, health professions rate, medical school entry
Strayhorn [22]	UNC MEDP (9 weeks)	Retrospective cohort	PB	Program ranking predicts medical school success
Wang et al. [23]	Michigan MMP (1 year)	Cross-sectional survey	UG	100% ↑ interest in medicine
Wei et al. [24]	Brain Camp (1 week)	Cross-sectional survey	HS	↑ healthcare interest
Winkleby [25]	Stanford SMYSP (5 weeks)	Retrospective cohort	HS	High college/graduation outcomes
Wrensford et al. [26]	Aetna HPPI (6 weeks)	Retrospective cohort	MS/HS	↑ graduation rates and SAT scores
Xirau-Probert et al. [27]	SHPEP (6 weeks)	Retrospective cohort	UG	Reduced barriers; ↑ motivation

**Table 2:** Risk of bias assessment of included studies using ROBINS-I tool Risk of bias was categorized as “Low,” “Moderate,” “Serious,” or “Critical” for each domain of the ROBINS-I V2 scale to assess bias risk in a standardized manner. A low risk of bias corresponds to little or no concern of bias, while a moderate risk of bias indicates some concern without it necessarily being an important risk of bias. From qualitative assessment and the scores given to each domain, a composite "Overall" risk of bias was assigned to each study. D1: Confounding; D2: Selection; D3: Classification; D4: Deviations; D5: Missing data; D6: Measurement; D7: Reporting bias; ROBINS-I: Risk of Bias in Non-randomized Studies of Interventions

Study	D1	D2	D3	D4	D5	D6	D7	Overall
Afghani et al. [4]	Moderate	Moderate	Low	Low	Low	Moderate	Low	Low
Bergstein et al. [5]	Moderate	Moderate	Low	Low	Low	Moderate	Low	Low
Crews et al. [6]	Moderate	Moderate	Low	Low	Low	Low	Low	Low
Danner et al. [7]	Moderate	Moderate	Low	Low	Low	Moderate	Low	Low
Douse et al. [8]	Moderate	Moderate	Low	Low	Low	Moderate	Low	Moderate
Giordani et al. [9]	Moderate	Moderate	Low	Low	Low	Low	Low	Low
Goldsmith, Tran, and Tran [10]	Moderate	Moderate	Low	Low	Low	Moderate	Low	Moderate
Hutchings et al. [11]	Moderate	Moderate	Low	Low	Moderate	Moderate	Low	Moderate
Krecko and Jung [12]	Moderate	Moderate	Low	Low	Moderate	Moderate	Low	Moderate
Papp et al. [13]	Moderate	Moderate	Low	Low	Moderate	Moderate	Low	Moderate
Rivers et al. [14]	Moderate	Moderate	Low	Low	Low	Moderate	Low	Moderate
Robinson et al. [15]	Moderate	Moderate	Low	Low	Low	Moderate	Low	Low
Rohrbaugh and Corces [16]	Moderate	Moderate	Low	Low	Low	Low	Low	Low
Salto et al. [17]	Moderate	Moderate	Low	Low	Low	Moderate	Low	Low
Satpathy et al. [18]	Moderate	Moderate	Low	Low	Moderate	Moderate	Low	Moderate
Schneid et al. [19]	Moderate	Moderate	Low	Low	Low	Low	Low	Low
Stephenson-Hunter et al. [20]	Moderate	Moderate	Low	Low	Low	Moderate	Low	Low
Stewart et al. [21]	Moderate	Moderate	Low	Low	Moderate	Low	Low	Low
Strayhorn [22]	Moderate	Low	Low	Low	Moderate	Low	Low	Low
Wang et al. [23]	Moderate	Moderate	Low	Low	Low	Moderate	Low	Low
Wei et al. [24]	Moderate	Moderate	Low	Low	Low	Moderate	Low	Low
Winkleby [25]	Moderate	Moderate	Low	Low	Low	Moderate	Low	Low
Wrensford et al. [26]	Moderate	Moderate	Low	Low	Low	Moderate	Low	Moderate
Xirau-Probert et al. [27]	Low	Moderate	Low	Low	Moderate	Moderate	Low	Low

Core Components in Early Exposure Programs to Medicine

The most common core components in early exposure programs were mentorship (n=18) and research (n=13). Outcomes most strongly associated with mentorship included increased further education (9 of 18 studies), increased biomedical and STEM degrees (7 of 18 studies), increased interest in medicine (4 of 18 studies), and greater self-efficacy (4 of 18 studies). By promoting close guidance with individuals with shared identity, students are more likely to develop the confidence, interest, and skills to pursue higher education [6,17,23]. Outcomes most strongly associated with research included increased pursuit of further education (7 of 13 studies) and increased attainment of biomedical and STEM degrees (6 of 13 studies). Research-focused programs for underrepresented undergraduate students may influence career decisions, self-confidence, and the intentions to pursue advanced education [12,15,17].

The primary participant education level was high school (n=14), followed by undergraduate (n=8), post-baccalaureate (n=4), and middle school (n=2).

Other Components in Early Exposure Programs to Medicine

Other components found in early exposure programs were observership experience (n=5), subspecialty exposure (n=5), multiprofession exposure (n=3), standardized exam preparation (n=2), and financial literacy (n=1). Outcomes most strongly associated with observership experience included increased further education (four of five studies) and increased biomedical and STEM degrees (three of five studies). Integrating clinical experience with mentorship may help stimulate interest in healthcare by providing firsthand experience [7,21,25]. Experiences beyond didactics, including anatomy practicals and shadowing, increased high school graduation rates, the pursuit of science degrees, and interest in health professions [7,21,25]. Outcomes most strongly associated with subspecialty exposure included increased interest in medicine (three of five studies) and increased further education (two of five studies). Programs targeting subspecialties can provide tailored information, preparation, and mentorship to foster interest tailored to a niche [5,8,11,18,24].

Duration of Treatment

Program durations varied widely: short (≤1 week, n=4), moderate (2-6 weeks, n=5), long (7-12 weeks, n=9), and extended (≥1 year, n=4). Notably, one study reported durations of one week for 5th graders, eight weeks for high school students, and 10 weeks for undergraduate students [6]. Krecko et al. conducted a prospective survey of eight United States high school-to-college programs funded by the Doris Duke Foundation, but did not specify the durations of these programs [12]. The remaining 22 programs ranged from one day to three years. Notably, while short programs demonstrated the ability to increase interest in medicine, enhance self-efficacy, reduce perceived barriers, and increase understanding of medical careers, certain outcomes were limited to programs with longer duration. Three of four programs associated with improved academic performance, seven of nine associated with increased further education, and six of seven associated with increased biomedical and STEM degrees were classified as long duration, with the remaining programs classified as moderate.

Outcomes of Early Exposure Programs to Medicine

The most frequently reported outcomes were further education (n=10), more students pursuing biomedical and STEM degrees (n=8), increased interest in medicine (n=7), improved academic performance (n=4), and greater self-efficacy (n=4).

Discussion

There is a substantial and heterogeneous body of literature evaluating early exposure to medical programs targeting underrepresented students. Several systematic and scoping reviews have synthesized efforts at later stages of medical training, such as interventions to increase the diversity of residency programs or to mentor students already enrolled in graduate medical education [3,31]. Other reviews have focused on identifying barriers and protective factors or on critiquing the methodology and quality of existing programs, noting flaws and challenges in their design, evaluation, and analysis [32]. Our review provides an evidence-based evaluation of the unique aspects of certain programs to extract lessons and examine the multifaceted impacts of these initiatives on student careers and diversity in medicine.

Barriers that hinder training success for underrepresented students include limited access to mentors with similar identities, insufficient institutional policies involving maternity leave or protected disabilities, stereotype threat (fear of confirming negative stereotypes about their group), imposter syndrome, and experiences of bias and discrimination [33]. A multifactorial array of financial, structural, social, and academic barriers poses challenges for underrepresented students. Systemic inequities that limit student recruitment and promotion, lack of comparable educational resources due to location or socioeconomic status, the financial burden of applications and examinations, and a lack of underrepresented role models all contribute to this situation [34]. Early exposure programs have provided opportunities for marginalized populations, enabled the development of research skills, fostered mentorship relationships, and cultivated interest in healthcare [34].

Mentorship was a key aspect in 18 of the 24 studies and was valued by participants. The University of California (UC) Summer Premed Program exemplifies the role of mentorship by assigning college and medical student coaches to underrepresented high school students to provide insight into medical school life [4]. High school students valued instructors with diverse cultural perspectives, while mentors reported improved leadership and teaching skills, greater appreciation for cultural diversity, and increased motivation for careers in academic medicine [7]. Numerous programs have incorporated peer and faculty mentorship components, demonstrating increased interest in healthcare, higher entry into medical and graduate school, and high student-reported satisfaction [6,17,23]. Moreover, program mentors, including medical students, residents, and physicians, can guide underrepresented students through the application process [11,16,20,24,25]. Ultimately, mentorship has demonstrated efficacy through interaction with others of similar identity, long-term guidance, and reduced perceived barriers to entering medicine.

Research played a role in early exposure programs in over half of the included studies (13 out of 24) and was associated with improved outcomes in shaping career trajectories, skills, networks, and interests. The Loma Linda University (LLU) Summer Health Disparities Research Program provided underrepresented high school and undergraduate students with mentorship, research experience, career development opportunities, and exposure to efforts aimed at reducing health disparities, resulting in significant improvements in research self-efficacy and skills. Hands-on research and mentorship were identified as the most valuable components of the program [17]. The National Institute of Diabetes and Digestive and Kidney Diseases (NIDDK) HS-STEP-UP 8-10-week summer program provided research exposure, including National Institutes of Health (NIH) symposium presentation opportunities, workshops, and longitudinal mentorship [14]. Therefore, integrating more research experiences into pipeline programs may cultivate multidisciplinary skills and foster interest in medicine.

Overall, four of the 24 studies, included in our systematic review with components such as mentorship, research, and multi-professional exposure, reported improved academic performance, which is critical for those who lack formal academic support or are first-generation college students with limited guidance. One of the earliest exposure programs was the Medical Education and Development Program (MEDP) at the University of North Carolina (UNC) Chapel Hill School of Medicine, which provided nine weeks of curriculum similar to the first-year medical school curriculum to underrepresented premedical students [22]. This study found that academic ranking within the MEDP predicted medical school academic outcomes, suggesting that premedical summer programs may predict medical school performance [22]. This may offer an opportunity for early intervention and academic support for students who are struggling [22]. One avenue of academic intervention may be post-baccalaureate programs, which have been shown to improve academic success among underrepresented students [9,19,22]. A University of Michigan (UMich) Medical School post-baccalaureate program offered one year of courses and research to underrepresented students before entering medical school [9]. Although students had lower mean undergraduate GPAs and MCAT scores than traditional students, all students demonstrated first-year medical school competence, suggesting that these programs may bridge disparities in prior education at the time of medical school entry [9]. These findings were corroborated by those of Schneid et al., who studied the effectiveness of the University of California San Diego (UCSD) School of Medicine’s seven-week prematriculation program, which provides biomedical instruction to underrepresented students [19]. Evidence of improved academic outcomes is not limited to the post-baccalaureate setting [26]. The Aetna Health Professions Partnership Initiative (Aetna HPPI) is a six-week academic enrichment program that provides underrepresented, low-income, and first-generation middle and high school students with educational sessions, standardized exam instruction, academic preparation, career advising, cultural activities, and mentoring [26]. This analysis found that all seniors graduated from high school with high college matriculation rates, and that SAT scores among program attendees were higher than the Hartford School District mean, suggesting that mentorship and academic support through pipeline programs may improve academic outcomes even during the high school phase [26].

Early exposure programs can promote broader exposure to medicine, including subspecialties (five out of 24 studies) and other healthcare professions (three out of 24 studies). Bergstein et al. conducted a survey of 259 female premedical students who received a Zoom panel featuring four female orthopedic surgeons discussing their experiences [5]. Interest in orthopedic surgery was greater among students who had connections to orthopedic surgeons, shadowed orthopedic surgery, or had undergone orthopedic surgery, highlighting the importance of prior connections and experience [5]. Following the panel, participants' interest in orthopedic surgery increased, and they were less likely to believe that gender would limit their opportunities, demonstrating the ability of subspecialty panels to alleviate perceived barriers [5]. A prominent surgery mentorship program evaluated by Hutchings et al. exposed underrepresented high school students to surgical subspecialties, with a focus on cardiothoracic surgery, through presentations, simulations, and discussions with diverse residents and medical students, application preparation, and internship opportunities [11]. Likert-scale data showed increases in confidence in college preparation, self-efficacy in pursuing surgery, and interest in pursuing medical careers [11]. Thus, such programs foster greater interest and promote improved self-confidence by making rigorous fields more approachable [11]. Similar programs involving observation, mentorship, preparation, and research have been established in otolaryngology, neurology, and regenerative medicine, enabling longitudinal subspecialty mentorship, opportunities for scholarly presentations, and increased interest in graduate and medical school, as well as STEM careers [8,18,24].

More recently, the authors implemented a pipeline program, Building Foundations, at the Medical College of Georgia to provide 46 undergraduate students from underrepresented backgrounds (80.4% female and 19.6% male; 45.7% Asian, 47.8% Black, 4.3% Hispanic, and 2.2% White) with early exposure to anesthesiology with support from the American Society of Anesthesiologists Committee on Professional Diversity. The program consisted of a one-day workshop incorporating hands-on skills training, didactic instruction, and the opportunity to interact/communicate with anesthesiologists, anesthesia residents, medical students and nurse anesthetists. This was followed by a structured longitudinal mentorship for participants who were mentored for one year by medical students, residents, and faculty. Participants were also offered opportunities to shadow in the operating room, completed by 17 students. Program outcomes were assessed using a 13-item Likert-scale survey, with responses analyzed using Mann-Whitney-Wilcoxon tests. Participants reported high satisfaction and perceived benefits, including strong endorsement of the program, increased interest in anesthesiology, and perceived gains in mentorship, networking, and medical school readiness. Although not part of the formal systematic review evidence, these preliminary unpublished findings convey an illustrative example and suggest that structured, mentorship-based early-exposure initiatives may be effective in supporting career development among students from underrepresented backgrounds.

Early exposure programs can provide multidisciplinary exposure to health professions beyond medicine (3 out of 24 studies). The Summer Health Professions Education Program (SHPEP) at the University of Florida was established to provide financial literacy, health policy, healthcare profession, and basic science knowledge, along with research and shadowing opportunities, for underrepresented undergraduate students in medicine, dentistry, pharmacy, or public health [27]. SHPEP demonstrates the role of such programs in exposing students to professions such as dentistry, pharmacy, and public health, enabling them to consider multiple career paths and make informed decisions [27]. Notably, SHPEP offered workshops and seminars on financial literacy and was the only program to emphasize personal finance [27]. Due to socioeconomic barriers faced by underrepresented, first-generation, and low-income groups, access to educational and financial resources, loan and scholarship opportunities, and understanding of personal finance are critical [27]. Goldsmith et al. evaluated an educational program that provided hands-on experiences for 28 underrepresented seventh-grade students by exposing them to pharmacy and physician assistant (PA) practitioners and laboratory skills [10]. Survey results showed increased knowledge of pharmacist and PA roles and heightened interest in health careers [10]. Such hands-on experiences may cultivate lasting interest in healthcare professions and promote greater understanding of career opportunities [10]. These findings are supported by a survey study conducted by Papp et al., which focused on 50 youth participants attending Asclepius Medical Camp and provided participants with information about medical careers, resulting in increased self-reported knowledge and quiz performance following the camp [13]. By promoting greater understanding of medical careers and opportunities, students may develop expectations and plans towards a profession while developing preparedness.

Overall, early exposure of underrepresented students to medicine has been shown to yield benefits, including increased interest in medicine, higher completion rates in biomedical and STEM degrees, greater understanding of career options, increased self-efficacy and confidence, reduced perceived barriers, and improved academic performance. The most frequently reported outcomes included increased interest in medicine, greater enrollment in biomedical and STEM degrees, and higher rates of further education. These outcomes, along with improved academic performance, were significantly associated with longer program duration, indicating that longer exposure to the medical field may be required to transform interest into lasting educational achievement. Mentorship and research were the most prominent components, with high student-reported value, and were associated with increased further education and biomedical/STEM degrees.

Multiple limitations exist for this review. One notable limitation is the heterogeneity of included programs, which employ various instructional approaches, durations, support methods, target demographics, and outcome measures, with reliance on short-term, self-reported outcomes, limiting comparability and longitudinal assessment. Given the substantial heterogeneity in program designs, participant populations, and outcome measures, the review relied on narrative synthesis because the data could not be meaningfully pooled for meta-analysis. Moreover, selection bias is present across multiple studies due to the recruitment of highly motivated students, who may already be more likely to achieve improved standardized test performance and pursue higher education independent of program participation. Ultimately, students applying to pipeline programs may have higher baseline interest in medicine than their peers, making it challenging to isolate program effects, especially with the lack of control groups limiting causality. Lastly, limited sample sizes and high program frequency in larger academic institutions limit the generalizability of findings.

Future directions for research including longitudinal studies assessing long-term career outcomes, and standardized outcome measures should be implemented across studies to allow for comparisons across programs and facilitate meta-analysis of program efficacy. Moreover, investigation into the relative effectiveness of individual program components is warranted, and assessing student perceptions of these components on perceived barriers and self-efficacy would yield novel insights, especially with greater longitudinal follow-up. Table 3 illustrates the frequency of core components, and Table 4 highlights the frequency of reported outcomes.

**Table 3:** Frequency of core components in early exposure programs to medicine Frequency represents the number of included studies reporting each core component.

Core Component	Frequency (n)	Studies
Mentorship	18	[4], [6], [7], [8], [11], [12], [13], [14], [15], [16], [17], [18], [20], [21], [23], [24], [25], [26]
Research	13	[6], [8], [9], [12], [14], [15], [16], [17], [18], [21], [23], [25], [27]
Application preparation	5	[11], [16], [20], [24], [25]
Observership experience	5	[7], [8], [21], [25], [27]
Subspecialty exposure	5	[5], [8], [11], [18], [24]
Multi-profession exposure	3	[10], [26], [27]
Standardized exam preparation	2	[16], [26]
Financial literacy	1	[27]

**Table 4:** Frequency of reported outcomes of early exposure programs to medicine Frequency represents the number of included studies reporting each outcome.

Reported Outcome	Frequency (n)	Studies
Increased further education	10	[6], [7], [8], [12], [16], [17], [18], [21], [25], [26]
Increased biomedical and STEM degrees	8	[6], [7], [12], [16], [17], [18], [21], [25]
Increased interest in medicine	7	[4], [5], [10], [11], [23], [24], [27]
Greater self-efficacy and confidence	4	[11], [12], [17], [20]
Improved academic performance	4	[9], [18], [22], [26]
Reduced perceived barriers	3	[5], [20], [27]
Greater understanding of medical careers	2	[10], [13]

## Conclusions

Early exposure programs for underrepresented students help diversify and promote interest in professions. Across early exposure programs, multiple themes emerge; however, mentorship was the most frequently reported component associated with positive outcomes, with participants reporting benefits from mentorship experiences across programs. When mentorship was integrated with research, the pairing was associated with improved outcomes in participants’ career trajectories, skill development, professional networks, and specialty interests. The use of mentorship to guide underrepresented students through graduate medical education has the potential to increase career self-efficacy. Four of the 24 studies included in this review reported improvements in academic performance, an especially meaningful outcome for students from marginalized backgrounds who may have limited access to academic support resources. The combination of mentorship and clinical experience in early exposure programs may stimulate interest in healthcare through firsthand experience. These programs have the potential to expose students to medical subspecialties and to health professions beyond medicine (e.g., dentistry, public health, pharmacy).

Overall, early exposure programs have demonstrated multifactorial benefits that may build interest in medicine, translating to preparedness and opportunities that underrepresented students have traditionally lacked. Mentorship and research were the most frequently featured components, with students reporting benefits from these components. Emerging research indicates that a more diverse student and physician population has positive effects on the healthcare system, and early exposure programs can help build a more diverse population and further progress within the healthcare complex.
